# Internet Addiction and Physical Activity among Polish and Portuguese Students in the Final Year of the COVID-19 Pandemic

**DOI:** 10.3390/jcm12165204

**Published:** 2023-08-10

**Authors:** Anna Zalewska, Monika Gałczyk, Marek Sobolewski, Hélder Fernandes

**Affiliations:** 1 Faculty of Health Sciences, University of Lomza, 14 Akademicka St., 18-400 Lomza, Poland; monikagalczyk@onet.eu; 2 Plant of Quantitative Methods, Rzeszow University of Technology, al. Powstancow Warszawy 12, 35-959 Rzeszow, Poland; mareksobol@poczta.onet.pl; 3 Health Sciences Research Unit: Nursing (UICISA: E), Instituto Politécnico de Bragança, 5300-253 Bragança, Portugal; helder@ipb.pt

**Keywords:** internet addiction, physical activity, Kimberly Young, IPAQ, students, Poland, Portugal

## Abstract

Aim: The purpose of this study was to analyze the level of Internet addiction and physical activity, as well as the relationship between internet addiction and physical activity, among Polish and Portuguese students in the final year of the COVID-19 pandemic. Methods: A web-based online survey was conducted among students in Poland and Portugal (398 respondents) aged between 17 and 26 in October 2022. The level of internet addiction was assessed by the Kimberly Young questionnaire in Polish and in Portuguese. The level of physical activity was measured by the international physical activity questionnaire in Polish and in Portuguese. Results: In the study population, the majority of the respondents were mildly addicted to the internet. The average level of addiction was reported by 9.3–23.5% of the respondents. The average level of internet addiction was higher among Portuguese students and among men. Those who had suffered from COVID-19 (especially more than once) and were more physically active showed a higher tendency towards internet addiction. Conclusions: Internet addiction is a problem that has to be monitored and given more attention, particularly among male students. The results presented provide the first statistical insight into the problem of internet addiction among students in both countries and form the basis for further studies. They also highlight the severity of the problem and point to the need for early preventive and protective interventions against problematic internet use. It appears that effective strategies should include promoting the benefits of physical activity.

## 1. Introduction

The threat of the COVID-19 pandemic has changed many areas of life, often making life more difficult and changing daily habits. The pandemic period even forced society to digitize selected areas of life several times, including studying, working or meeting friends. In this situation, it quickly became apparent that one of the most important tools for daily life was the internet [1,2]. It is primarily a source of information, knowledge and communication. The high availability of the internet on mobile devices is one of the main factors contributing to its excessive use [3]. The term addiction is defined as the body’s strong habit of taking unhealthy substances or performing a particular activity too often. In a modern world dominated by computers and smartphones, uncontrolled use of the internet can develop into an addiction [4,5] and not only have long-term effects physically but also become an obsession, leading to mental health problems [6,7,8].

On the one hand, the progressive spread and popularization of digital technology in several spheres of life aids individuals in navigating daily life, but on the other, it also introduces some limits. These restrictions may lead to a decrease in physical activity, which would lower quality of life [9,10].

Systematic physical activity at an appropriate level has many benefits for health and wellbeing. It has a beneficial effect on the cardiovascular, respiratory, nervous and musculoskeletal system [11,12]. Numerous scientific studies show that as little as 10 min of daily activity such as walking or cycling can affect a person’s mood [13,14,15]. There is also evidence in the literature that the negative effects of reduced physical activity on the body contribute to a more rapid onset of some diseases of civilization, such as back pain, obesity, diabetes or hypertension [16,17,18].

Young people with high levels of physical activity tend to spend less time on stationary activities such as the internet. A number of studies have shown that misuse of the internet has a number of negative consequences for an individual’s health [19,20]. There are also studies in the literature that describe associations between levels of internet addiction and physical activity [21,22,23,24].

Researchers from the University of Economics in Katowice conducted a study in 2020, which showed the growing importance of the internet to society. According to this research, in 2020, 91% of households in the European Union (including Poland) had access to the internet and used it systematically. In comparison, only 70% of households in the European Union were connected to the internet in 2010. The results show that internet access still varies from country to country. The highest levels were recorded for 2020 in the Netherlands, Finland and Germany, and the lowest in Bulgaria, Greece and Portugal. Four member states (Denmark, Finland, Sweden and the Netherlands) recorded the highest percentage of regular internet users, over 90%. A high percentage of at least 80% of people using the internet every day was registered in 14 countries. The EU average for 2020 was 80%, and thirteen member states were below this average, including Poland with a score of 72%. Only Portugal, Greece and Romania performed worse than Poland [25].

However, despite the risks of frequent internet use to important areas of life including interpersonal relationships as well as physical or mental health, the number of people using the internet continues to rise [26,27,28,29,30]. Therefore, there is a need for multidimensional research into the factors that influence the negative effects of internet addiction. This may in the future contribute to the development of educational programs to prevent the spread of this harmful phenomenon.

The aim of this study was to provide a preliminary assessment of the level of internet addiction and physical activity and the relationship between internet addiction and physical activity among Polish and Portuguese students in the final year of the COVID-19 pandemic.

## 2. Materials and Methods

### 2.1. Participants and Procedure

A cross-sectional survey of Polish and Portuguese students was conducted in October 2022. The survey was made available on the internet through a link to Google Forms with a questionnaire created by the authors. It was distributed through the researchers’ online e-learning platforms and social media. It targeted students from a variety of disciplines. In addition to the questionnaires, the link included information about voluntary consent to participate in the survey and its anonymity. The questionnaire was prepared in two languages (Polish and Portuguese). The requirements for participation in the study were: student status, consent to participate in the study, residence in Poland or Portugal and complete completion of the questionnaire. The exclusion criteria were: no student status, no consent to participate in the study, residence in a country other than Poland and Portugal and incomplete completion of the questionnaire. The researchers received a total of 502 responses to their request, of which 398 correctly completed questionnaires were analyzed (229 from Poland and 169 from Portugal). Respondents were between 17 and 26 years old, and the median for age in both countries was 22 years. There were 247 women and 151 men in the study group.

The project was approved by the University of Lomza Senate Committee on Ethics in Scientific Research. Participation in the study was voluntary, and the findings were published (Journal of Laws 2018, item 1000) in accordance with Regulation (European Union) 2016/679 of the European Parliament and of the Council of 27 April 2016, on the protection of natural persons with regard to the processing of personal data and on the free movement of such data and repealing Directive 95/46/EC, in the Personal Data Protection Act of 10 May 2018. GDPR, or the general data protection regulation, the study’s objectives, poll methodology and pertinent data protection requirements were all disclosed to respondents.

### 2.2. Methods for Assessing Internet Addiction and Physical Activity

#### 2.2.1. Kimberly Young Questionnaire

Internet addiction was assessed using the Kimberly Young questionnaire [31]. This is a 20-item instrument that assesses frequency of internet use. The respondents answered questions on a five-point scale [24]. A minimum of 20 and a maximum of 100 points could be obtained. The more points the respondents scored, the higher their level of internet dependence. The respondents were divided into three groups: low dependence (20–49 points), medium dependence (50–79) and high dependence (80–100 points) [24]. The Cronbach’s alpha value for the Kimberly Young questionnaire reported in the literature is >0.7 [32,33,34].

#### 2.2.2. International Physical Activity Questionnaire

An abbreviated version of the international physical activity questionnaire (IPAQ), designed for individuals aged 15–69 years, was used to assess physical activity levels [35]. Its questions cover all forms of daily physical activity. It assesses activity in and around the home, at work and during leisure time. The respondents answered questions about the amount of uninterrupted time of at least 10 min spent sitting, walking and engaging in vigorous and moderate physical activity. The activities assessed were reported in units of MET -min/week, i.e., the product of a factor assigned to a given activity and the number of days in the week that this activity was performed as well as the duration per day in minutes [35]. The Cronbach’s alpha value for the international physical activity questionnaire reported in the literature is >0.7 [36,37,38].

### 2.3. Statistical Methods

The Statistica v. 13 software was employed for the statistical analysis (TIBCO Software Inc., Palo Alto, CA, USA, 2017). The significance of the differences in internet addiction and physical activity level (IPAQ) between groups of Portuguese and Polish students was assessed using the Mann–Whitney test, and differences in the percentage distribution of internet addiction level classification and activity level were assessed using the chi-squared test of independence. Internet addiction and physical exercise were correlated using Spearman’s rank correlation coefficient. The significance of differences in internet addiction versus activity level classification was assessed using the Kruskal–Wallis test. The use of non-parametric techniques was required due to the non-normality of the distributions of the IPAQ and internet addiction measures, both of which exhibited very substantial right-handed asymmetry. All statistical analyses were conducted using a significance level of *p* < 0.05 (*); however, results for *p* < 0.01 (**) and *p* < 0.001 (***) were also mentioned.

## 3. Results

### 3.1. Comparative Characteristics of the Studied Communities

The analysis was based on data collected from a group of Polish and Portuguese students consisting of 398 people (Table 1). Both groups were comparable in terms of age, with female students outnumbering males in both groups. Respondents were between 17 and 26 years old (Portugal) and between 18 and 26 years (Poland). The median for age in both countries was 22 years.

**Figure 1:**
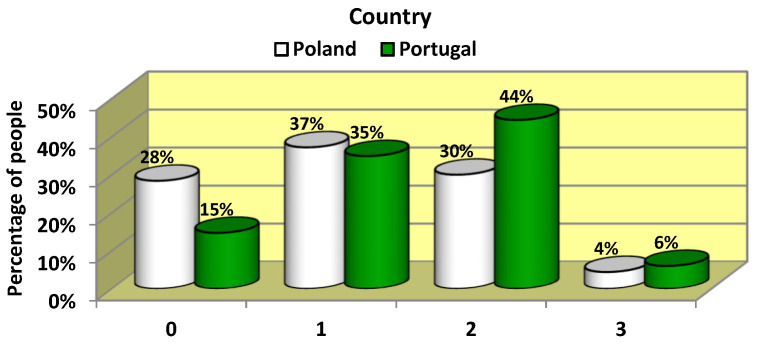
COVID-19 disease.

The declaration of having COVID-19 was reported by more than 70% of respondents among Poles and almost 85% among Portuguese students (Figure 1). The difference in the proportion of COVID-19 sufferers among the respondents was statistically significant (*p* = 0.0029). However, the researchers had no method of determining the dependability of the responses.

### 3.2. Comparison of Internet Addiction among Students from Both Countries

Considering the total population of Polish and Portuguese respondents, it was concluded that the average level of internet addiction was higher among the Portuguese adolescents (Table 2).

### 3.3. Comparison of Internet Addiction by Student Sex

A factor that may influence internet addiction levels is undoubtedly sex. Information on the extent of internet addiction in both countries, broken down by sex, is summarized below.

As can be seen, the level of internet addiction was higher among the males in both countries, with a slightly larger sex difference among the Portuguese students (Table 3). When comparing the two countries, the Portuguese students were more addicted to the internet among both males and females. However, the difference was not statistically significant for women (*p* = 0.1074), while it was significant for men (*p* = 0.0437 *).

On the other hand, if the results are classified as low, medium and high, it shows that the majority of the individuals were mildly addicted to the internet (about 75–90%), and there was no statistically significant difference between the Polish and Portuguese students, although more individuals in the latter country showed a medium or high (mostly medium) severity of internet addiction (Table 4).

### 3.4. COVID-19 Incidence and Internet Addiction

The condition of COVID-19 may be associated with forced isolation and as such may influence an increased frequency of internet use. On the other hand, there are so many factors that interfere with this potential correlation that one would tend to expect no correlation here either.

Interestingly, there was some correlation between the number of reported COVID-19 cases and internet addiction (Table 5). These were very weak correlations that were only statistically significant for the Polish women, while they were almost statistically significant for the male groups (*p* < 0.10). However, it is possible to speak of some correlation and this is an interesting conclusion. A positive sign of the correlation coefficient means that people who suffered from COVID-19 (especially more than once) were more likely to become internet addicts.

### 3.5. Comparison of Activities among Students from Both Countries

The classifications of the IPAQ measures are presented below (Table 6). It can be seen that the Polish women were more extreme in terms of physical activity, while more than half of the Portuguese women reported a medium level of physical activity.

### 3.6. Internet Addiction and Physical Activity

Statistically significant correlations were also found between physical activity and internet addiction (Table 7). Positive correlations were observed in all the groups considered, but statistically significant correlations were found among the Portuguese women and Polish men. These were weak correlations, as is usually the case in studies based on participants’ subjective responses and complex questionnaires on behavior or mental states. What is surprising and somewhat troublesome from an interpretation point of view, however, is the direction of the correlations—the correlation coefficients were positive, which means that those who were more physically active also showed more internet dependence.

## 4. Discussion

Internet addiction is a growing social problem at a public health level that affects people worldwide. It is particularly dangerous in adolescent and student populations [39]. At the same time, it is one of the fastest growing addictions worldwide, negatively affecting the physical and mental health of young people [40]. Looking holistically, during the first wave of the COVID-19 pandemic, mild and more severe symptoms of internet addiction were diagnosed in about one third of students in Poland [41].

At the same time, students in Portugal were found to use social media for between 3 and 8 h per day, with this time increasing during isolation [42]. In an international comparison, Thai students reported a lower prevalence of internet addiction during the pandemic at 5.8% [43], while other studies have found a much higher prevalence of this worrying phenomenon. More specifically, the results are as follows: 10.4% of students addicted to the internet were found in Mexico [44], 12.4% in Spain [45], 16.8% in the United States [46], 20% in Brazil [47], 28.4% in China [48], 32.6% in Bangladesh [49], 51.7% in Egypt [40] and 55% in Nigeria [50]. At the same time, the level of physical activity also decreased significantly during the pandemic period, both among young people from Poland [51,52] and Portugal [53,54] but also among students worldwide [55].

The majority of respondents in the sample population reported a minor level of internet addiction. However, a worrying conclusion was that, on average, 9.3–23.5% of the respondents showed signs of addiction. Similar findings were obtained from a study of university students in Slovakia and the Czech Republic, where the majority of participants showed a modest level of internet addiction. However, the study’s findings on mild addiction revealed lower values, with 3.4% of Czech and 6% of Slovak students reporting mild internet addiction [56].

Sex is clearly a factor that could affect the severity of internet addiction. It is a significant danger factor for internet addiction [57]. In our study, males in both nations had a greater level of internet addiction; however, the Portuguese students had a slightly larger gender gap. The Czech Republic and Slovakia [56], Italy [58,59], the United States [60], Iran [61], Turkey [62], Poland [63], Tanzania [64] and India [65], among other countries, all found that male students were more likely to be addicted to the internet. These results confirm the findings obtained before the COVID-19 pandemic [66]. Of course, there are also studies in the literature in which women relied more heavily on boarding schools [40,67] or equally as much as men [68], but these are rare. According to the literature, the primary virtual activity for male students with moderate to severe internet addiction is online gaming, and for female students it is online streaming [69].

The average level of internet addiction was higher in young students from Portugal. The discrepancy found between the countries studied requires further investigation. It is known that students from urban areas are more likely to be affected by internet addiction [70]. Urban areas have better access to the internet, and their residents use it more often and are more likely to be addicted [71].

In both Poland and Portugal, the students who had suffered from COVID-19 (especially more than once) were more likely to be addicted to the internet. In studies conducted during the lockdown in the Dominican Republic, Egypt, Guyana, India, Mexico, Pakistan and Sudan, it was observed that students who reported COVID-19-related symptoms had sleep disturbances and higher levels of internet addiction [72].

The correlation coefficients of internet addiction and physical activity found in this study were positive, which means that individuals who were more physically active were also more addicted to the internet. It seems that the cause of this correlation is due to another common factor, but it is not evident from the data collected in this study. Perhaps it is a general “life activity” during the pandemic—some people may have fallen into a general apathy and then “did not want” to do anything either real or virtual. This could also be due to the fact that during the pandemic, various online platforms, apps and videos promoting physical activity were used more frequently. At the same time, they facilitated and promoted regular exercise without leaving the house. This trend could also be observed after the pandemic in Poland and Portugal. When interpreting the results, it should also be taken into account that the people surveyed were mostly not highly addicted to the internet (85% were categorized as “low” in terms of their level of internet addiction), so a slightly higher level of addiction in physically active people does not necessarily already indicate a pathological condition. These findings are surprising, as the data available in the literature clearly suggest that physical activity reduces the risk of developing certain psychiatric disorders and is an effective adjunct to the therapeutic process for depression, anxiety and addictions [73].

The authors acknowledge the limitations of the presented study. The first is the nature of the study (cross-sectional), which does not provide robust causal evidence for the observed associations. Other limitations include the location of the study—the internet—the sample size, and the use of self-report questionnaires. The study does have strengths, however, particularly in terms of the findings on the problem of internet addiction among university students in Poland and Portugal, where this topic is poorly researched and has little evidence base. The authors also consider the use of validated research instruments, the low cost of conducting the study and the ease of access to the study group as strengths.

## 5. Conclusions

Internet addiction among adults is a phenomenon of recent years, made more evident by the COVID-19 pandemic. The results of this study are important because it is a problem that has to be monitored and given more attention, particularly among students. The findings of this study close a significant research gap and can be regarded as innovative, particularly in Portugal. The results presented provide the first statistical insight into the problem of internet addiction among students in both countries and form the basis for further studies. They also highlight the severity of the problem and point to the need for early preventive and protective interventions against problematic internet use. It appears that effective strategies should include promoting the benefits of physical activity (despite the findings of our own study). The next step could be to develop educational programs to prevent the spread of this harmful phenomenon. This study also clearly indicates (what is important in daily practice) that prevention programs should target male students in particular.

## Figures and Tables

**Table 1:** Sex of the surveyed students.

Sex	Country		Total
	Poland	Portugal	
Women	129 (56.3%)	118 (69.8%)	247
Men	100 (43.7%)	51 (30.2%)	151
Total	229	169	398

**Table 2:** Internet addiction among students from both countries.

Country	Measure of Internet Addiction (*p* = 0.0398 *)						
	Mean	Median	Std. Dev.	c_25_	c_75_	Min	Max
Poland	34.6	31	12.6	25	40	20	84
Portugal	38.6	34	16.0	27	46	20	83

*p*-test probability value calculated using the Mann–Whitney test, *p* < 0.05 (*).

**Table 3:** Internet addiction in both countries by sex of students.

Country	Measure of Internet Addiction									
	Women (*p* = 0.1074)					Men (*p* = 0.0437 *)				
	Mean	Median	Std. Dev.	c_25_	c_75_	Mean	Median	Std. Dev.	c_25_	c_75_
Poland	33.2	30	12.4	25	39	36.5	34.5	12.7	27	42
Portugal	36.8	33	15.2	26	42	42.7	40	17.0	31	51

*p*-test probability value calculated using the Mann–Whitney test, *p* < 0.05 (*).

**Table 4:** Categorized level of internet addiction in both countries by sex of students.

Level of Internet Addiction on the Internet	Sex			
	Women (*p* = 0.1452)		Men (*p* = 0.1855)	
	Poland	Portugal	Poland	Portugal
Low	116 (89.9%)	96 (81.4%)	84 (84.0%)	38 (74.5%)
Average	12 (9.3%)	21 (17.8%)	16 (16.0%)	12 (23.5%)
High	1 (0.8%)	1 (0.8%)	0 (0.0%)	1 (2.0%)

*p*-test probability value calculated by means of the chi-squared independence test.

**Table 5:** COVID-19 disease and internet addiction.

Sex	COVID-19 Disease and Internet Addiction	
	Poland	Portugal
Women	0.19 (*p* = 0.0302 *)	0.11 (*p* = 0.2282)
Men	0.19 (*p* = 0.0572)	0.23 (*p* = 0.0992)

*p* < 0.05 (*).

**Table 6:** Sex classification of IPAQ measures for both countries.

Activity Level	Sex			
	Women (*p* = 0.0000 ***)		Men (*p* = 0.6395)	
	Poland	Portugal	Poland	Portugal
High	55 (42.6%)	32 (27.1%)	59 (59.0%)	26 (51.0%)
Average	30 (23.3%)	67 (56.8%)	30 (30.0%)	18 (35.3%)
Low	44 (34.1%)	19 (16.1%)	11 (11.0%)	7 (13.7%)

*p*-test probability value calculated by means of the chi-squared independence test, *p* < 0.001 (***).

**Table 7:** Internet addiction and measures of physical activity.

IPAQ	Measure of Internet Addiction	
	Poland	Portugal
Women		
Intense effort	0.17 (*p* = 0.0539)	0.27 (*p* = 0.0036 **)
Moderate effort	0.08 (*p* = 0.3585)	0.11 (*p* = 0.2388)
Walking	0.04 (*p* = 0.6346)	0.25 (*p* = 0.0072 **)
Total effort	0.12 (*p* = 0.1864)	0.22 (*p* = 0.0164 *)
Men		
Intense effort	0.23 (*p* = 0.0191 *)	0.23 (*p* = 0.1103)
Moderate effort	0.18 (*p* = 0.0756)	0.22 (*p* = 0.1296)
Walking	0.18 (*p* = 0.0753)	0.14 (*p* = 0.3430)
Total effort	0.21 (*p* = 0.0367 *)	0.21 (*p* = 0.1357)

Spearman’s coefficient of correlation (with assessment of its statistical significance), *p* < 0.05 (*), *p* < 0.01 (**), IPAQ—international physical activity questionnaire.

## Data Availability

The data that support the findings of this study are openly available in RepOD at https://doi.org/10.18150/0J3FKR (accessed on 14 February 2023).
